# CRISPR-Cas-Based Pen-Side Diagnostic Tests for *Anaplasma marginale* and *Babesia bigemina*

**DOI:** 10.3390/microorganisms12122595

**Published:** 2024-12-15

**Authors:** Robert Muriuki, Maingi Ndichu, Samuel Githigia, Nicholas Svitek

**Affiliations:** 1 Department of Veterinary Pathology and Parasitology, Faculty of Veterinary Medicine, University of Nairobi, Nairobi P.O. Box 30197, Kenya; r.muriuki@cgiar.org (R.M.); nmaingi@uonbi.ac.ke (M.N.); sgithigia@uonbi.ac.ke (S.G.); 2 Health Program, International Livestock Research Institute (ILRI), Nairobi P.O. Box 30709, Kenya

**Keywords:** babesiosis, anaplasmosis, pen-side test, point-of-care diagnostics, CRISPR-Cas powered diagnostics

## Abstract

*Anaplasma marginale* and *Babesia bigemina* are tick-borne pathogens, posing significant threats to the health and productivity of cattle in tropical and subtropical regions worldwide. Currently, detection of *Babesia bigemina* and *Anaplasma marginale* in infected animals relies primarily on microscopic examination of Giemsa-stained blood or organ smears, which has limited sensitivity. Molecular methods offer higher sensitivity but are costly and impractical in resource-limited settings. Following the development of a pen-side test for detecting *Theileria parva* infections in cattle, we have created two additional CRISPR-Cas12a assays targeting *Anaplasma marginale* and *Babesia bigemina*. The assays target the major surface protein 5 (MSP5) for *A. marginale* and rhoptry-associated protein 1a (RAP1a) for *B. bigemina*. These additional tests involve a 20 min recombinase polymerase amplification (RPA) reaction followed by a 60 min CRISPR-Cas12a detection with a lateral strip readout. Results demonstrate high specificity, with no cross-reactivity against other tick-borne parasites, and a limit of detection down to 10^2^ DNA copies/µL of each target marker. The findings pave the way for sensitive and user-friendly pen-side tests to diagnose *A. marginale* and *B. bigemina* infections.

## 1. Introduction

We recently developed a rapid pen-side test for *Theileria parva*, the causative agent of East Coast fever (ECF), using the RPA/CRISPR-Cas technologies [1]. In our desire to design better tools for the differential diagnosis of a number of other tick-borne diseases that share clinical signs, we aimed to increase our portfolio of rapid CRISPR-Cas pen-side tests targeting two additional tick-borne pathogens, *Babesia bigemina* and *Anaplasma marginale*.

These pathogens cause babesiosis and anaplasmosis, respectively, both of which are important tick-borne diseases of cattle worldwide. Bovine babesiosis is a globally distributed tick-borne protozoan disease caused by pathogenic species such as *Babesia bovis*, *B. bigemina*, and *B. divergens*. Clinically the disease manifests by fever, anemia, hemoglobinuria, and splenomegaly, resulting in the death of the animal [2]. In Africa, including Kenya, bovine babesiosis is caused by *Babesia bigemina* and *Babesia bovis*. *Babesia bigemina* is more widespread, while *B. bovis* is more critical and pathogenic, and they are both transmitted by *Rhipicephalus* ticks [3]. Another major tick-borne bacterial disease in cattle is bovine anaplasmosis, caused by intracellular bacteria known as *Anaplasma* and is mainly transmitted by *Rhipicephalus* (*Boophilus*) *microplus* ticks [4]. The causative agents in this genus are *A. marginale*, *A. centrale*, *A. phagocytophilum*, and *A. bovis*. *Anaplasma marginale* infects red blood cells and is highly pathogenic in cattle with a wide distribution in tropical and subtropical regions [5]. In young calves, *A. marginale* causes persistent fever, anemia, jaundice, lethargy, and weight loss, while in adults it causes abortion in pregnant animals, decreased milk yield in lactating animals, and death in over 50% of untreated animals [6]. *Babesia bigemina* and *A. marginale* cause mortalities and morbidities leading to losses in the production of milk, meat, and other livestock by-products. Consequently, they cause severe economic losses to livestock farmers involved in dairy and beef production in tropical and sub-tropical regions [3].

Diagnosis of these diseases has mostly relied on clinical signs [7], which is not confirmatory because most clinical signs are shared among most tick-borne diseases (TBDs) and microscopic examination of Giemsa-stained blood smears [8]. However, microscopy is relatively insensitive, and it is difficult to identify the organisms to the species level [9]. Serological methods have been applied for the detection of antibodies [10,11], but they do not show current infection. Molecular methods have been exploited for the detection of these infections, such as the reverse line blot hybridization assay [12] and the nested polymerase chain reaction (nPCR) [13,14], which have proved to be more sensitive and specific in the diagnosis of these diseases, discriminating to the species level. However, the limitation with these tests is that they require expensive equipment and specialized labor, making them unreachable to farmers in resource-limited areas that need these tests.

In our previous study [1], we developed an RPA/CRISPR-Cas12a-based pen-side assay for detecting *T. parva* in cattle. This assay demonstrated the ability to identify eight *T. parva* field strains, with a detection limit of one infected cell per 3 µL of blood. Initially, the assay was optimized using PCR as a pre-amplification step and a flow cytometry readout. To enhance field applicability, the method was then adapted to include an RPA pre-amplification step and a lateral flow strip readout. Following a similar development process, this current study describes the creation of two additional RPA/CRISPR-Cas12a-based diagnostic assays targeting the *A. marginale* major surface protein 5 (*msp5*) and *B. bigemina* rhoptry-associated protein 1a (*RAP1a*) genes.

## 2. Materials and Methods

**Figure 1:**
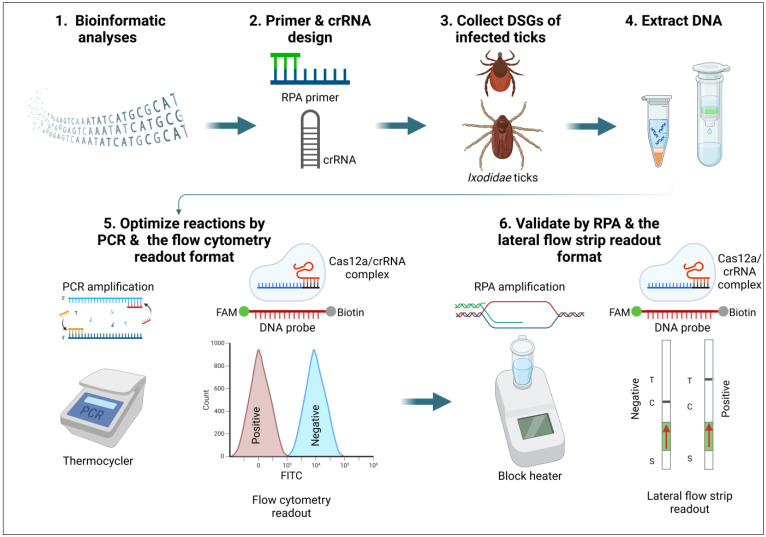
Schematic representation of the steps taken in the development of the assays. Created in BioRender. Svitek, N. (2024) BioRender.com/a88f007.

The assays developed in this study were designed and optimized in a similar way as previously described in [1] and the steps summarized below (Figure 1).

### 2.1. Bioinformatics Analysis

The major surface protein 5 (*msp5*) and the rhoptry-associated protein 1a (*RAP1a*) genes were used as target markers for *A. marginale* and *B. bigemina*, respectively, due to their previous use in the molecular detection of these pathogens [5,15]. Therefore, three complete coding sequences (CDS) of the *Anaplasma marginale msp5* (accession numbers AY714547.1, ON456134.1, and ON456135.1) and three partial coding sequences of *Babesia bigemina RAP1a* (accession numbers KP893330.1, KP347558.1, and KP347559.1) were downloaded from the National Center for Biotechnology Information (NCBI). A multiple sequence alignment was carried out with these *A. marginale* and *B. bigemina* genes to identify the conserved regions in both genes. The conserved regions were then used to design CRISPR RNA (crRNA) targets as well as polymerase chain reaction (PCR) and recombinase polymerase assay (RPA) primers used for pre-amplification.

### 2.2. CRISPR RNA (crRNA) and RPA/PCR Primer Design

The crRNAs and primers design followed the methods described in [1]. Synthesis and purification of the crRNAs were carried out by Integrated DNA Technologies (IDT, Coralville, IA, USA). Four crRNAs, with two per gene, were designed and used in this study as shown in Table 1.

Two pairs of primers per target marker (F1 and R1 and F2 and R2) were designed as shown in Table 2, amplifying amplicon sizes of 108 and 122 base pairs (bp), respectively, for *A. marginale* and 202 and 181 bp, respectively, for *B. bigemina.* Each amplicon was targeted by a single crRNA. Later the primers were mixed to incorporate dual crRNAs per target by combining the forward primer of set 1 (F1) with the reverse primer of set 2 (R2) of each target marker. These modified primer sets amplified amplicon sizes of 414 and 399 bp for *A. marginale* and *B. bigemina*, respectively. Synthesis and purification of the primers were carried out by Macrogen Incorporated (Amsterdam, The Netherlands). The RPA primers designed also served as PCR primers. The designed sequences for both *A. marginale* and *B. bigemina* were run on the NBCI BLAST platform to confirm their specificity to each parasite.

### 2.3. DNA Preparation

The DNA used for assay optimization was extracted from tick salivary glands infected with either *Anaplasma marginale* or *Babesia bigemina*. Assay specificity was tested using DNA from other tick-borne pathogens, such as *Theileria parva*, *Theileria mutans*, and *Theileria lestoquardi*.

### 2.4. PCR and RPA Pre-Amplification of msp5 and RAP1a Gene

For specificity testing, PCR was performed in a 25 µL reaction mixture consisting of 3 µL of the DNA template, 3 µL of 10X PCR buffer, 2.4 µL of 10 µM of dNTPs mix, 5 µL of 25 mM MgCl_2_, 1.5 µL of 10 nM of each primer, and 1.5 units of DNA polymerase (Sigma Aldrich, St. Louis, MO, USA). The amplification protocol consisted of an initial denaturation step at 95 °C for 1 min, followed by 35 cycles of 94 °C for 10 s, 55 °C for 20 s, and 72 °C for 30 s, with a final extension at 72 °C for 5 min.

To assess the sensitivity of the assays, a high-fidelity PCR was performed to generate high-quality amplicons. The PCR was set up in a final volume of 25 µL, containing 12.5 µL of Q5^®^ High-Fidelity 2X DNA Polymerase from New England Biolabs (Ipswich, MA, USA, Cat No. M0515), 2.5 µL of 10 nM of each primer, and 5 µL of DNA template. The amplification protocol was carried out as follows: an initial denaturation step at 98 °C for 30 s was followed by 35 cycles of 98 °C for 10 s, 55 °C for 20 s, and 72 °C for 30 s, with a final extension at 72 °C for 5 min. The amplicons were purified using the Zymo DNA Clean and Concentrator Kit from Zymo Research (Irvine, CA, USA, Cat. No. D4033). The purified amplicons were viewed on a 1.5% agarose gel. The copy numbers of these purified amplicons were determined using the formula below:

$$\mathrm{Number}\;\mathrm{of}\;\mathrm{copies}\;(\mathrm{molecules})=\frac{X\;\mathrm{ng}*6.0221\times 10^{23}\;\mathrm{molecules}/\mathrm{mole}}{{\left(N*660\;g/\mathrm{mol}\right)}^{\;+}*1\times 10^{9}\;\mathrm{ng}/g},$$

where X = amount of amplicon (ng), N = length of dsDNA amplicon, 660 g/mol = average mass of 1 bp dsDNA, 6.022 × 10^23^ = Avogadro’s constant, and 1 × 10^9^ = conversion factor.

After determining the copy numbers, dilutions were prepared ranging from 10^10^ to 10^0^ copies/µL for each target. One microliter of each dilution was used as a template for both PCR and RPA preamplification. All the assays were first optimized with PCR as a preamplification step and flow cytometry as the readout before switching to RPA and lateral flow strip readout (Figure 1).

The RPA reaction mixture was performed in a 50 µL reaction system using the commercially available TwistAmp basic kit (TwistDx, Cambridge, UK). A master mix (44.5 µL) consisting of 29.5 µL of rehydration buffer, 2.4 µL of each primer (forward and reverse primers, 10 μM), and 10.2 µL of nuclease-free water was added to the lyophilized pellet and mixed by gentle pipetting. Three microliters of DNA template were added to the reaction tubes. Then, 2.5 µL of magnesium acetate (280 mM) was placed on the tube lid. The tubes were gently closed and centrifuged. These tubes were immediately incubated at 39 °C for 20 min. For the sensitivity assays, the DNA template was reduced to 1 µL.

### 2.5. CRISPR-Cas12a Detection

The *Lachnospiraceae bacterium* Cas12a (*Lba* Cas12a) trans-cleavage assays for the single crRNA approach were performed as described previously [1]. The reaction was carried out in a 30 µL reaction volume. Firstly, the Cas12a ribonucleoprotein was formed by preparation of a master mix that included 2 µL of *Lba* Cas12a (500 nM, New England Biolabs, M0653T), 3 µL of crRNA 1 (500 nM), 3 µL of 10X NEBuffer™ r2.1, and 18 µL of nuclease-free water and pipetted into reaction tubes. The tubes were incubated at 25 °C for 15 min, after which 1 µL of ssDNA fluorescent probe (500 nM) and 3 µL of pre-amplified DNA template were added. The reaction was mixed gently and spun and immediately incubated at 37 °C for 1 h. The dual crRNA approach *Lba* Cas12a assays were performed in a similar manner but adjusted slightly by replacing 3 µL of nuclease-free water with an equal volume of crRNA 2 (500 nM).

### 2.6. Flow Cytometry Assay

The assay was carried out as previously described in [1]. The data analysis was performed by calculating the fluorescence intensity ratio using the following formula: MFI_beads+probe_/MFI_sample_. All graphs were generated with the GraphPad Prism 10.3.1 software.

### 2.7. Lateral Flow Strip Assay

To read the detection result using the lateral flow assay, 10 µL of the sample to be analyzed was added to 100 μL of the HybriDetect assay buffer and mixed gently. A lateral flow strip was placed into the mixture and incubated for 3 min at room temperature. After which they were removed, and results interpreted immediately. A sample was considered positive when the test band (upper band) appeared significantly stained, while negative samples only had the control line (lower band) visibly stained.

## 3. Results

### 3.1. The Single crRNA (crRNA1)/CRISPR-Cas12a Assay Is Highly Specific

**Figure 2:**
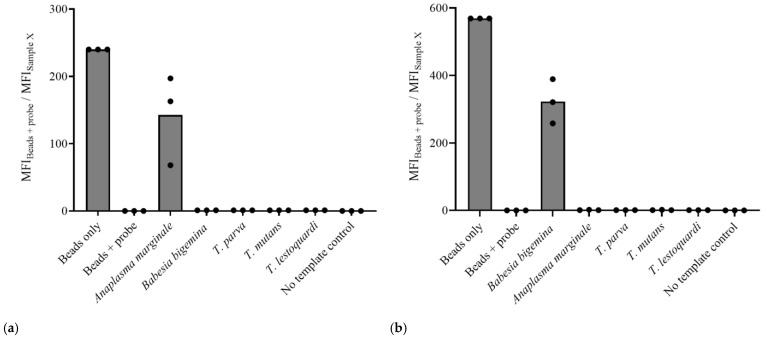
Histogram representation of the flow cytometry-based readout for the specificity of the PCR/Cas12a assays using the single crRNA approach for (**a**) *A. marginale* and (**b**) *B. bigemina*.

To ensure the specificity of the Cas12a-based assays for *A. marginale* and *B. bigemina*, we tested these PCR-Cas12a reactions using the DNA from these parasites as well as the *Theileria* species *T. parva*, *T. mutans*, and *T. lestoquardi*. The specificity was tested using a single crRNA per target. Data obtained from the single crRNA approach showed that only *A. marginale* and *B. bigemina* samples demonstrated trans-cleavage of the fluorescent probe, while the same was not observed in the *Theileria* species (Figure 2).

### 3.2. The Single crRNA (crRNA 1)/CRISPR-Cas12a Assay Demonstrates Fair Sensitivity

**Figure 3:**
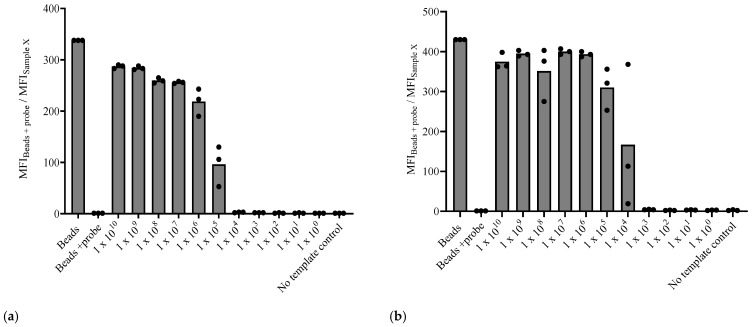
Flow cytometry readout for the sensitivity of the PCR-Cas12a assays. (**a**) Sensitivity of the *A. marginale* assay with a single crRNA (1), (**b**) Sensitivity of the *B. bigemina*-specific test with a single crRNA (1).

The limit of detection for both *A. marginale* and *B. bigemina* assays was determined using purified PCR amplicons with the flow cytometry readout and using a single crRNA approach. The *A. marginale*-specific test was able to detect up to 10^5^ DNA copies of the *msp5* gene per µL, while the *B. bigemina*-specific test was slightly more sensitive with a detection limit of 10^4^ DNA copies of the *RAP1a* gene per µL (Figure 3).

### 3.3. The Dual crRNA/CRISPR-Cas12a Assay Is Highly Specific

**Figure 4:**
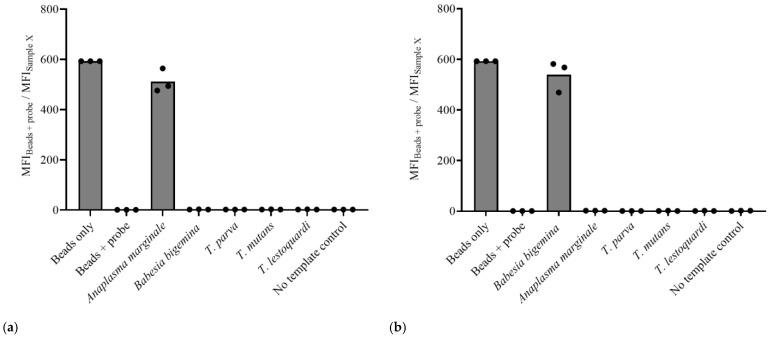
Histogram representation of the flow cytometry-based readout for the specificity of the PCR-Cas12a assays using dual crRNAs for (**a**) *A. marginale* and (**b**) *B. bigemina*.

Since the assay using a single crRNA was not sensitive enough, we decided to add an additional crRNA, targeting a larger amplicon, to each test to improve their sensitivity. The first step was to assess the specificity of the PCR-Cas12a dual crRNA assay for each test. The results with the dual crRNA were consistent with those observed using the single crRNA, as again only the *A. marginale* or the *B. bigemina* samples elicited the trans-cleavage of the FAM-biotin probe when their respective primer pairs and crRNAs were used (Figure 4).

### 3.4. The Dual crRNA/CRISPR-Cas12a Assay Demonstrates Enhanced Sensitivity

**Figure 5:**
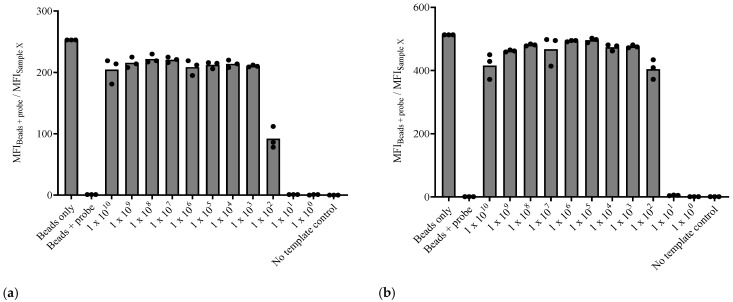
Flow cytometry readout for the sensitivity of the PCR-Cas12a assays using two crRNAs per target gene. (**a**) Sensitivity of the *A. marginale* assay with dual crRNAs, (**b**) sensitivity of the *B. bigemina*-specific test with dual crRNAs.

The sensitivity of the assays using dual crRNAs per target was then tested. A significant increase from 10^2^- to 10^3^-fold in assay sensitivity with both tests was observed. The limit of detection for both tests improved to 10^2^ DNA copies per μL for each target gene (Figure 5).

### 3.5. Developing a Field Deployable-Based Lateral Flow-Based Assay

**Figure 6:**
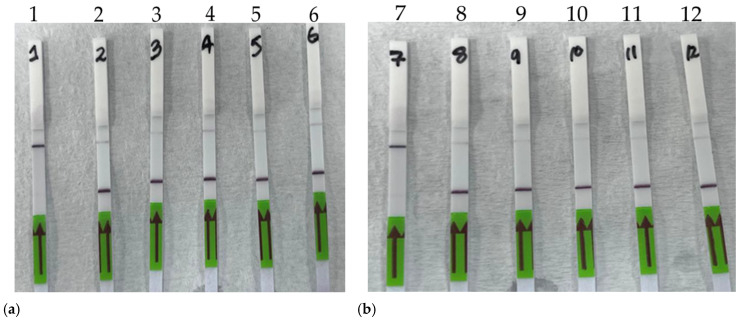
Specificity of the RPA-Cas12a assay for *Anaplasma marginale* and *Babesia bigemina* using the lateral flow readout format. (**a**) Lateral flow assay specificity for *A. marginale*: 1. *A. marginale* positive sample, 2. *B. bigemina*, 3. *T. parva*, 4. *T. mutans*, 5. *T. lestoquardi*, and 6. no template control. (**b**) Lateral flow strip assay specificity for *B. bigemina*: 7. *B. bigemina positive sample*, 8. *A. marginale*, 9. *T. parva*, 10. *T. mutans*, 11. *T. lestoquardi*, and 12. no template control.

After optimizing the assays using the PCR pre-amplification step and a flow cytometry-based readout, we transitioned to using RPA reactions for the pre-amplification step. Additionally, the readout format was changed from flow cytometry to lateral flow strips to enhance the test’s ease of use in the field or on farms. Based on the results from both specificity and sensitivity assays, the dual crRNA approach was chosen for the lateral flow-based assay. The specificity of the RPA-Cas12 assay was first evaluated, showing consistent results with those obtained with the PCR-Cas12a assay, confirming the specificity of the *A. marginale* and *B. bigemina* tests following this change in protocol (Figure 6).

**Figure 7:**
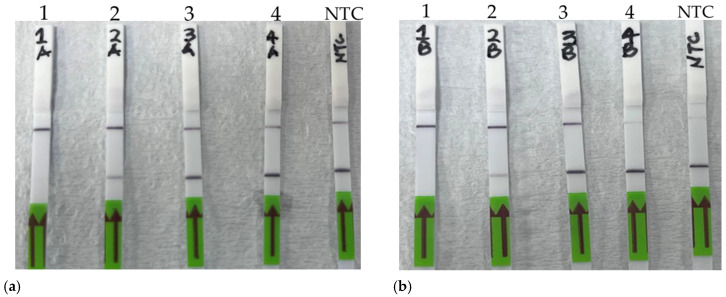
Sensitivity of the RPA-Cas12a assay for *Anaplasma marginale* and *Babesia bigemina*. (**a**) Lateral flow assay limit of detection for *Anaplasma marginale*: 1. 10^3^ DNA copies/µL, 2. 10^2^ DNA copies/µL, 3. 10^1^ DNA copies/µL, 4. 10^0^ DNA copies/µL, and NTC: no template control. (**b**) Lateral flow strip sensitivity for *B. bigemina*: 1. 10^3^ DNA copies/µL, 2. 10^2^ DNA copies/µL, 3. 10^1^ DNA copies/µL, 4. 10^0^ DNA copies/µL, and NTC: no template control.

Finally, the sensitivity of the RPA-Cas12a assays for both *A. marginale* and *B. bigemina* was tested. Due to the previously acquired flow cytometry results and the availability of lateral flow strips, the sensitivity of the RPA-Cas12a assays was tested with DNA dilution ranging from 10^3^ to 10^0^ copies/µL. Results show that both *A. marginale* and *B. bigemina*-specific tests were able to detect again up to 10^2^ DNA copies of the target gene per µL, although both tests seemed to cleave 10^2^ copies/µL partially (Figure 7).

## 4. Discussion

New CRISPR-Cas12a-based assays coupled with RPA and lateral flow strip readouts for the detection of *Anaplasma marginale* and *Babesia bigemina* infections are described. The tests were first optimized with a PCR pre-amplification step and flow cytometry readouts. Subsequently, they were adapted for field compatibility by using RPA and lateral flow strips to create a more field-friendly diagnostic tool. The developed assays demonstrate high specificity and sensitivity that can be applied in the control and management of these infections.

The *Anaplasma marginale*-specific test is based on the major surface protein 5 (MSP5), due to its conserved nature [16], and has thus been used to develop other molecular assays such as a quantitative real-time PCR [17] and a semi-nested PCR for detecting anaplasmosis infections in carrier animals [5]. The CRISPR-Cas12a assay based on the *msp5* gene exhibited high specificity, especially when tested against commonly occurring tick-borne parasites such as *B. bigemina*, *T. parva*, *T. mutans*, and *T. lestoquardi*. The results were consistent when using a single or dual crRNA approach and regardless of the readout method. The assays demonstrated higher sensitivity using dual crRNA reactions as compared to using a single crRNA targeting the same genes. The dual crRNA approach enhanced the limit of detection (LOD) by three logs to 10^2^ DNA copies/µL. This limit of detection is within the range of other CRISPR-Cas-based tests achieving similar LODs as our assay [18,19,20].

**Figure 8:**
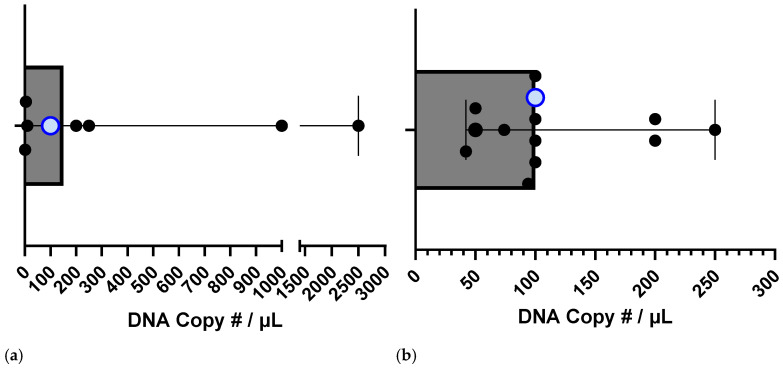
Sensitivity comparison between our test and other tests. (**a**) Sensitivity of other diagnostic tests for *Anaplasma marginale* and *Babesia bigemina* in comparison to our tests (light blue dot) [references taken from Table 3 where data of copy numbers was available], and (**b**) other CRISPR-Cas diagnostic tests that use copy numbers as a measure of limit of detection: 42 copies/µL [20], 50 copies/µL [31], 50 copies/µL [32], 74 copies/µL [33], 94 copies/µL [34], 100 copies/µL [35], 100 copies/µL [36], 100 copies/µL [37], 100 copies/µL [38], 200 copies/µL [39], 200 copies/µL [40], and 250 copies/µL [41].

When comparing the sensitivity and specificity of our CRISPR-Cas assays with already existing tests for both *A. marginale* and *B. bigemina*, our test falls below the sensitivity median of *A. marginale* and *B. bigemina* specific tests (Table 3, Figure 8a) and on the sensitivity median value compared to other developed CRISPR-Cas-based assays (Figure 8b).

The use of two or more crRNAs has been employed in several CRISPR-Cas12a-based assays, leading to improved sensitivity. Similar studies have also observed enhanced sensitivity when using multiple crRNAs [21,30]. As we worked on developing and optimizing our *A. marginale*-specific CRISPR/Cas12a assay, we noted that Sutipatanasomboon et al. [21] had just recently described a similar assay but targeting another gene, the major surface protein 4, using a fluorescent or colorimetric lateral flow dipstick readout.

In regard to *B. bigemina*, the rhoptry-associated protein 1a (*RAP1a*) is a highly conserved gene that has been utilized in several molecular diagnostic test developments [3,15,23,24]. The *RAP1a*-based Cas12a assay we developed showed no cross-reactivity when tested against related tick-borne pathogens, including *T. mutans*, *T. parva*, *T. lestoquardi*, and *A. marginale*. The *B. bigemina*-specific test again demonstrated higher sensitivity when targeting a larger amplicon of 399 bp using two crRNAs.

The PCR/RPA primer sequences and CRISPR RNAs used in this study were analyzed for specificity using the BLAST platform. All sequences from *Anaplasma marginale* aligned precisely with its major surface protein 5. Similarly, *Babesia bigemina* sequences showed the same level of specificity. This specificity is critical, especially for CRISPR RNAs, as even slight mismatches can impair the trans-cleavage activity of Cas12a enzymes. We successfully avoided such mismatches. For both species, the top 100 BLAST hits corresponded exclusively to *A. marginale* and *B. bigemina*. Moreover, this was also confirmed experimentally for *B. bigemina* with closely related apicomplexan parasites.

When the pre-amplification step was modified from PCR to RPA, and the readout format was switched to lateral flow-based strips, the RPA-Cas12a assay remained highly specific and sensitive, with a sensitivity comparable to that of the assay using PCR and flow cytometry for data acquisition. The *B. bigemina*-specific RPA-Cas12a described in this study is the first of its kind, as there is currently no pen-side diagnostic for bovine babesiosis.

As these are innovative tests, further research is necessary to assess their performances using a larger set of samples from both infected ticks and infected animals in parallel to the already existing molecular diagnostic tests for these diseases. Additionally, clinical validation with blood samples will be of critical importance in establishing the clinical effectiveness of these diagnostic tests. Future work will focus on having a single multiplex test that can differentially diagnose anaplasmosis, babesiosis, and East Coast fever by combining these individual tests with a recently developed test by our team for the detection of *T. parva* in cattle [1].

In summary, this study demonstrates that the CRISPR-Cas12a tests that we developed are highly specific, with no cross-reactivity observed with other related pathogens, and sensitive tools that offer an alternative method for the detection of *A. marginale* and *B. bigemina*. These tests are easy to perform as well as rapid, delivering results in less than two hours. With their ability to facilitate on-site diagnosis or field-based point-of-care testing, these tests hold significant potential for the control and management of these two important tick-borne diseases.

## Figures and Tables

**Table 1:** CRISPR RNA (crRNA) sequences specific towards *msp5* and *RAP1a* genes used in this study.

CRISPR RNA	Sequence	Length
*msp5* crRNA 1	rUrArA rUrUrU rCrUrA rCrUrAr rArGrU rGrUrA rGrArU rCrArG rCrArA rArArU rCrGrG rCrGrA rGrArG rGrU	41
*msp5* crRNA 2	rUrArA rUrUrU rCrUrA rCrUrA rArGrU rGrUrA rGrArU rGrArU rGrCrG rArGrA rArUrU rCrArG rArUrG rCrU	41
*RAP1a* crRNA 1	rUrArA rUrUrU rCrUrA rCrUrA rArGrU rGrUrA rGrArU rUrUrG rUrUrA rGrCrU rUrGrU rUrGrA rArGrA rArG	41
*RAP1a* crRNA 2	rUrArA rUrUrU rCrUrA rCrUrA rArGrU rGrUrA rGrArU rGrGrU rArUrC rCrArG rArArG rGrCrG rUrUrG rArA	41

**Table 2:** Sequences of MSP5 and RAP1a recombinase polymerase amplification (RPA) primers designed and used in the assay to detect *Anaplasma marginale* and *Babesia bigemina*, respectively.

Primer	Sequence	Length
*msp5* F1	GCCGTGTTCCTGGGGTACTCCTATGTGAACAA	32
*msp5* R1	AGACGCGGAGGCTATGCCCTCACTTACAACTT	32
*msp5* F2	CTGTTGATCCGAAAAATGACACCGTAGCCAAGC	33
*msp5* R2	CTTGTAGTTTTCAACCAGGCTCTTTATGTCTGC	33
*RAP1a* F1	GACGCTGCCTTCATGCTTTTCAGGGAAAGTGA	32
*RAP1a* R1	ACAACGTAGTCATGTAGAAGTACTGCGATGCG	32
*RAP1a* F2	GACCGTTGACTTTACGGCGGCTAAGTTCTTCA	32
*RAP1a* R2	CATCATGTACTCGCCGTAGCCGCTAGCTATTT	32

**Table 3:** Comparison of the sensitivity and specificity of existing diagnostic tests for *A. marginale* and *B. bigemina*.

Title	Assay and Gene Target	Sensitivity and Specificity	Reference
***Anaplasma marginale***			
RPA-CRISPR/Cas12a assay for the diagnosis of bovine *Anaplasma marginale* infection	RPA-CRISPR-Cas12a assay, major surface protein 4 (MSP4)	Sensitivity of 4 copies/µL of *msp4* gene No cross-reactivity observed when tested with DNA *from Babesia bovis*, *T. orientalis*, and *T. evansi*	[21]
Specific molecular detection and characterization of *Anaplasma marginale* in Mongolian Cattle	Nested PCR based on the *msp5* gene	Sensitivity: limit of detection was 200 copies/µL of the *msp5* gene No cross-reactivity when tested against *Ehrlichia canis*, *E. muris*, *Ehrlichia* sp., *Anaplasma bovis*, *A. centrale*, *A. platys*, *Anaplasma* sp. closely related to *A. phagocytophilum* of Japan, *A. phagocytophilum*, *Theileria orientalis*, *Babesia bovis*, and *B. ovata*	[14]
Molecular detection of *Anaplasma marginale* infection in carrier cattle	Semi-nested PCR, major surface protein 5	Sensitivity limit of detection of 30 infected erythrocytes per ml of blood No cross-reactivity when tested against *Theileria annulata*, *Babesia bigemina*, and *Trypanosoma evansi*	[5]
Real-time PCR assay with an endogenous internal amplification control for detection and quantification of *Anaplasma marginale* in bovine blood	TaqMan Quantitative PCR, based on major surface protein 1 (*msp1α*) gene	Sensitivity: Able to detect up to 1 copy of the *msp1* gene No cross-reactivity observed when tested with closely related *Anaplasma* spp.: *A. centrale*, *A. bovis*, *A. phagocytophilum*, *A. ovis-positive*, and *A. platys*	[22]
Detection and quantification of *Anaplasma marginale* DNA in blood samples of cattle by real-time PCR	TaqMan-based real-time PCR assay based on the *msp1b* gene	Sensitivity: 10^1^ DNA copies of the msp1b gene and 30 *Anaplasma*-infected erythrocytes mL^−1^ of blood No cross-reactivity with other pathogens, including *A. centrale*, *A. bovis*, *A. ovis*, *A. phagocytophilum*, *B. bovis*, *B. bigemina*, *T. annulata*, and *T. buffeli*	[23]
Comparison of three nucleic acid-based tests for detecting *Anaplasma marginale* and *Anaplasma centrale* in cattle	Three nucleic acid tests for *A. marginale* based on the *msp1b* gene RLB, nested PCR, and qPCR	Sensitivity: 2500 copies of the *msp1β* gene for RLB, 250 copies of the same gene by nPCR and qPCR No cross-reactivity when tested against *Anaplasma* sp., *A. phagocytophilum*, *B. bovis*, and *Theileria parva*	[24]
CRISPR-Cas-based pen-side diagnostic tests for *Anaplasma marginale* and *Babesia bigemina*	RPA-Ca12a assay based on *msp5* gene	Sensitivity: 100 copies/µL of *msp5* gene No cross-reactivity when tested against *T. parva*, *T. mutans*, *Babesia bigemina*, and *T. lestoquardi DNA*	Our test.
*Babesia bigemina*			
Molecular detection and identification of *Babesia bovis* and *Babesia bigemina* in cattle in northern Thailand	Nested PCR based on RAP1a gene for *B. bigemina*	Sensitivity: The detection limit was equivalent to a parasitemia of 0.00000001% No cross-reactivity when tested against DNA from *B. bovis*, *T. orientalis*, *T. gondii*, and *N. caninum*	[25]
A quantitative PCR assay for the detection and quantification of *Babesia bovis* and *B. bigemina*	SYBR green qPCR based on the *cytochrome B* gene	Sensitivity of 1000 copies, translating to 0.1 fg of DNA No cross-reactivity observed when tested against *T. annulata*, *T. buffeli*, *T. equi*, and *B. caballi*	[26]
Development of TaqMan-based real-time PCR assays for diagnostic detection of *Babesia bovis* and *Babesia bigemina*	TaqMan assay based on the 18S rRNA	The sensitivity of the test is at 2.5 parasites/µL of infected blood No cross-reaction observed when tested against DNA from *Theileria parva*, *Trypanosoma evansi*, and *Neospora caninum*	[27]
Rapid and sensitive detection of *Babesia bovis* and *Babesia bigemina* by loop-mediated isothermal amplification combined with a lateral flow dipstick	A LAMP-LFP assay based on the *cytochrome B* gene	Sensitivity of 0.8 g of *Babesia bigemina* DNA No cross-reactivity with DNA from *Babesia bovis*, *Theileria sergenti*, *Theileria ovis*, *Theileria equi*, and *Toxoplasma gondii*	[28]
Development and standardization of a loop-mediated isothermal amplification (LAMP) test for the detection of *Babesia bigemina*	LAMP technique based on the *ama-1* gene	Sensitivity of 0.00000001% of parasitemia Highly specific with no cross-reactivity observed when tested with DNA from *B. bovis*, *Anaplasma marginale*, *A. phagocytophilum*, *A. centrale*, *Trypanosoma theileri*, *Bos taurus*, *Homo sapiens*, *Rhipicephalus microplus*, and *Neospora caninum*	[29]
CRISPR-Cas-based pen-side diagnostic tests for *Anaplasma marginale* and *Babesia bigemina*	RPA-Ca12a assay based on *RAP1a* gene	Sensitivity: 100 copies/µL of *RAP1a* gene No cross-reactivity when tested against *T. parva*, *T. mutans*, *Babesia bigemina*, and *T. lestoquardi DNA*	Our test.

## Data Availability

The raw flow cytometry data (mean fluorescence intensities) can be accessed through the Supplementary Materials file.

## Supplementary Materials

The following supporting information can be downloaded at: https://www.mdpi.com/article/10.3390/microorganisms12122595/s1, Figure S1. Heat map representation of the mean fluorescence intensities for Anaplasma marginale specificity using a single crRNA approach; Figure S2. Heat map representation of the mean fluorescence intensities for Anaplasma marginale specificity using a dual crRNA approach; Figure S3. Heat map representation of the mean fluorescence intensities for Anaplasma marginale sensitivity using a single crRNA approach; Figure S4. Heat map representation of the mean fluorescence intensities for Anaplasma marginale sensitivity using a dual crRNA approach; Figure S5. Heat map representation of the mean fluorescence intensities for Babesia bigemina specificity using a single crRNA approach; Figure S6. Heat map representation of the mean fluorescence intensities for Babesia bigemina specificity using a dual crRNA approach; Figure S7. Heat map representation of the mean fluorescence intensities for Babesia bigemina sensitivity using a single crRNA approach; Figure S8. Heat map representation of the mean fluorescence intensities for Babesia bigemina sensitivity using a dual crRNA approach.
