# Supplementary material for: CRISPR-Cas-Based Pen-Side Diagnostic Tests for Anaplasma marginale and Babesia bigemina
Source: Microorganisms. 2024 Dec 15;12(12):2595. doi: 10.3390/microorganisms12122595 (PMC11678693; doi:10.3390/microorganisms12122595)
Supplement: Supplementary file 1 [file microorganisms-12-02595-s001.zip › microorganisms-3179623-supplementary.pdf]

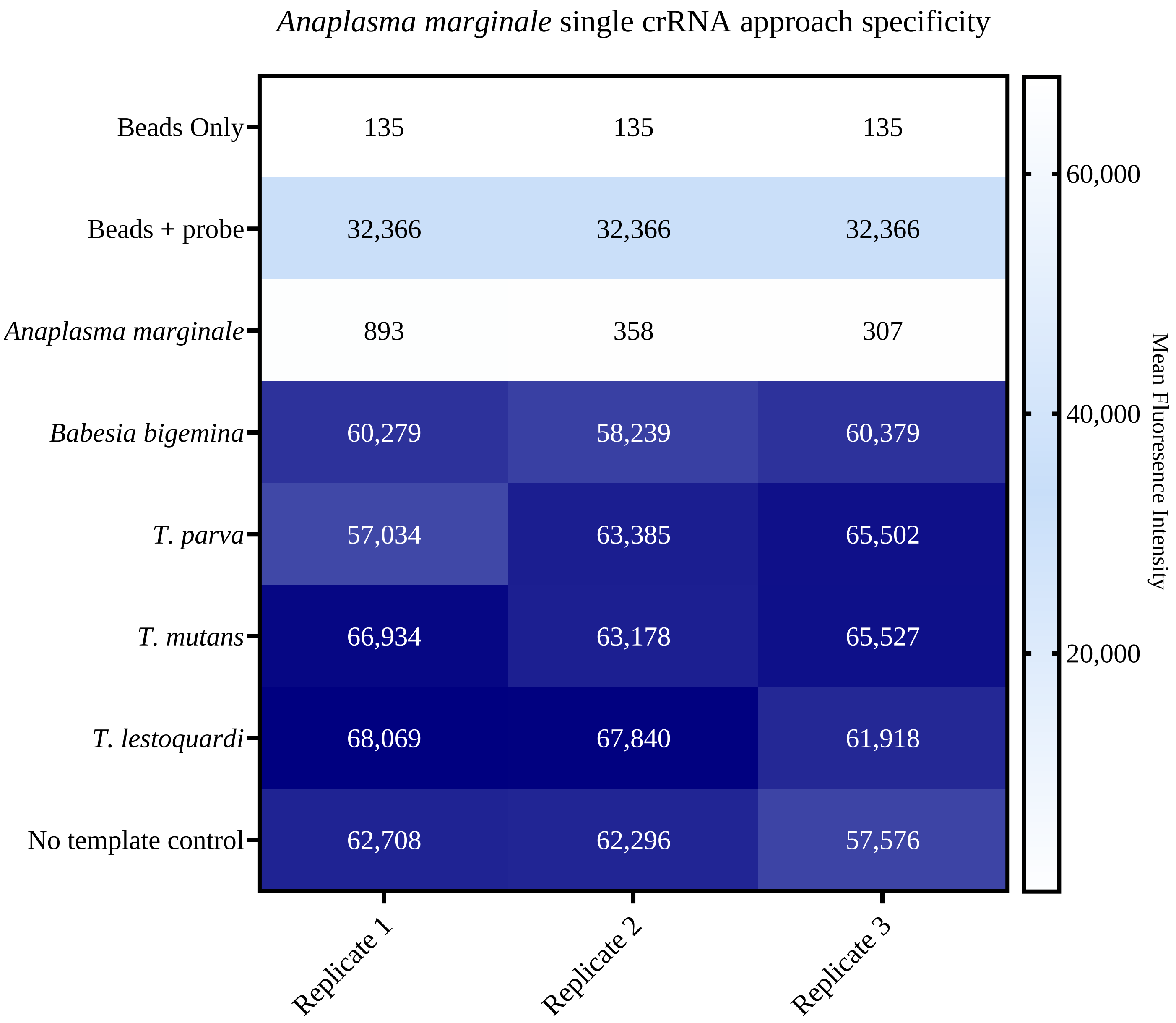

Sup. Fig. S1. Heat map representation of the mean fluorescence intensities for *Anaplasma marginale* specificity using a single crRNA approach

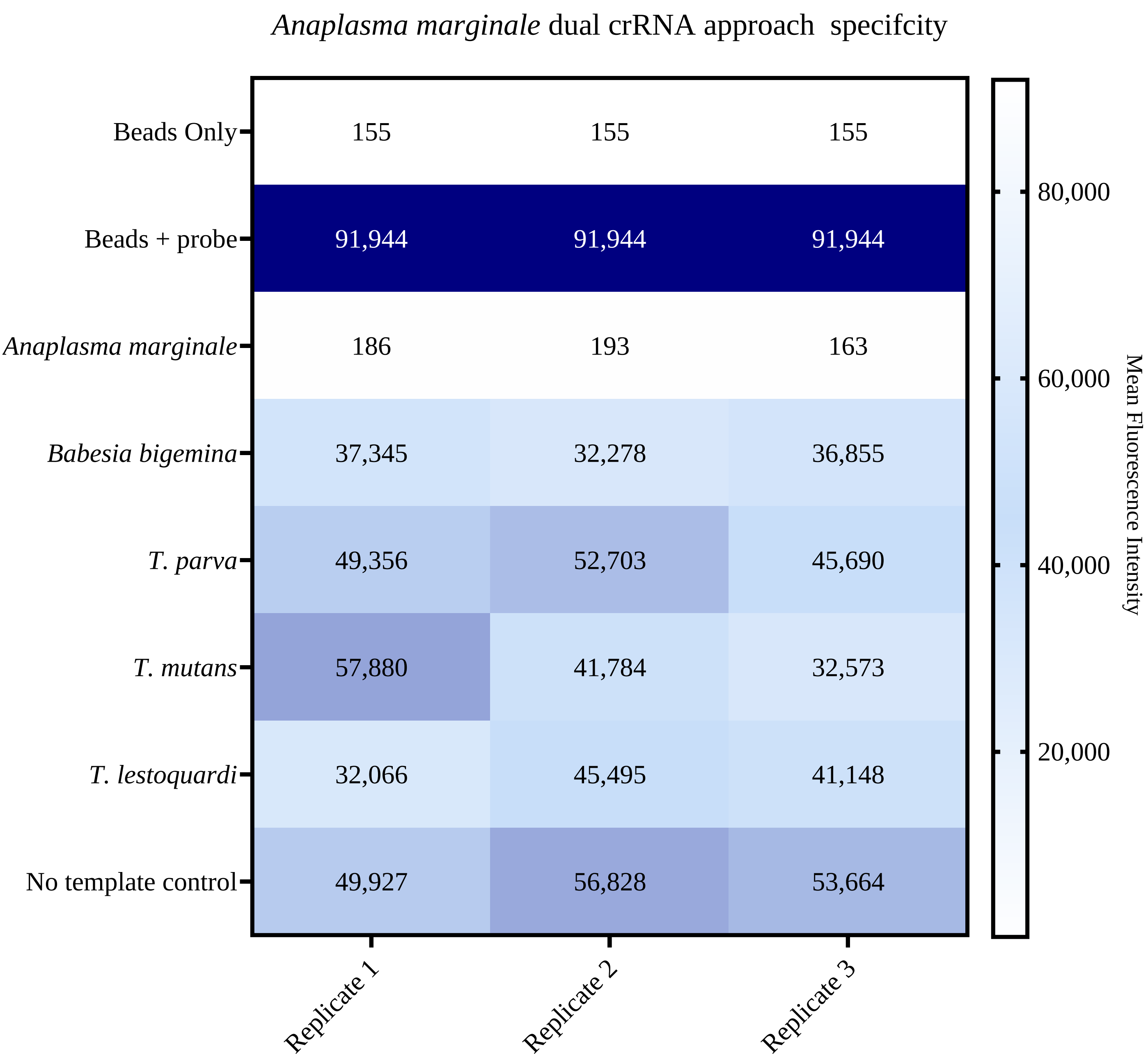

Sup. Fig. S2. Heat map representation of the mean fluorescence intensities for *Anaplasma marginale* specificity using a dual crRNA approach

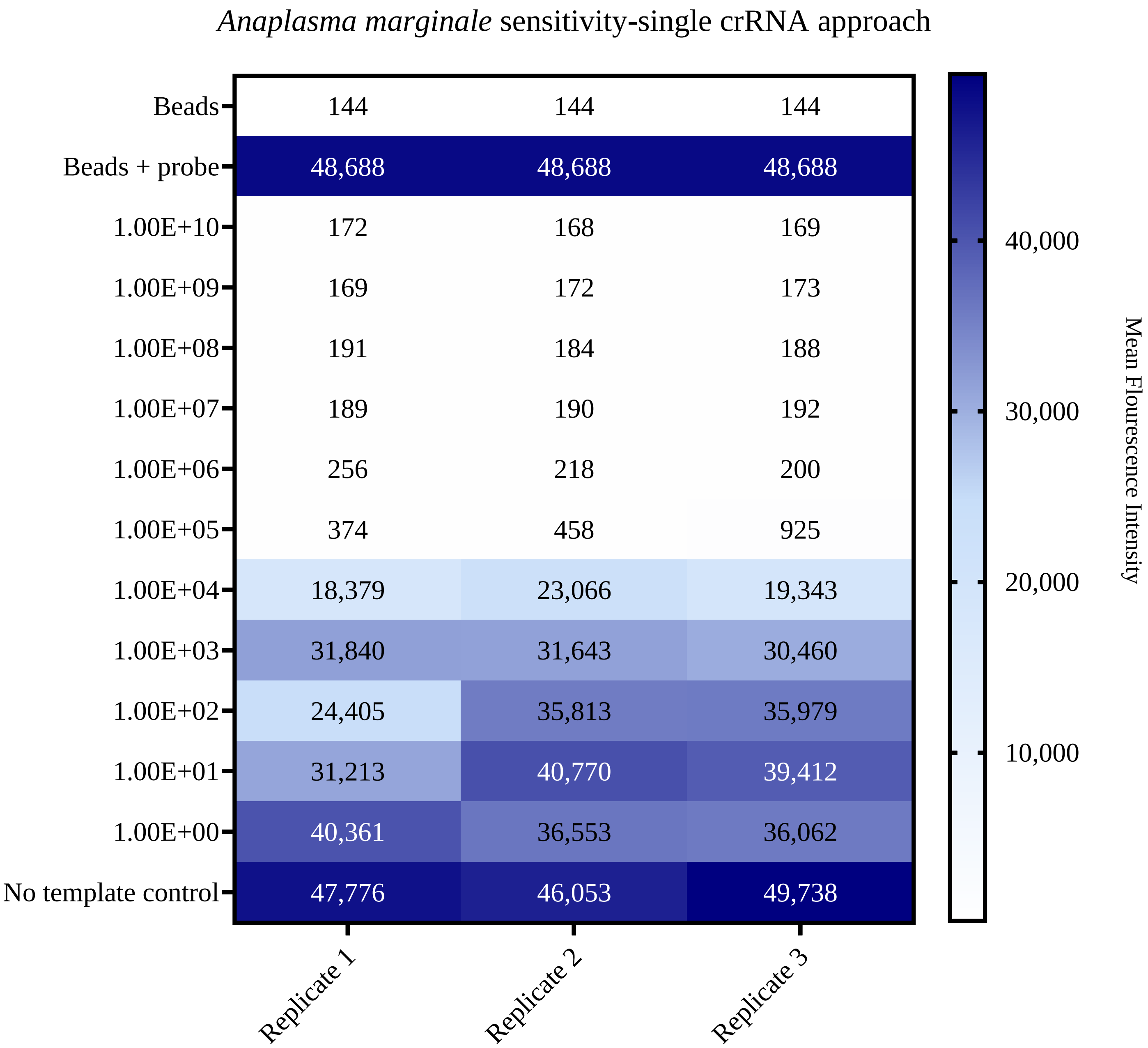

Sup. Fig. S3. Heat map representation of the mean fluorescence intensities for *Anaplasma marginale* sensitivity using a single crRNA approach

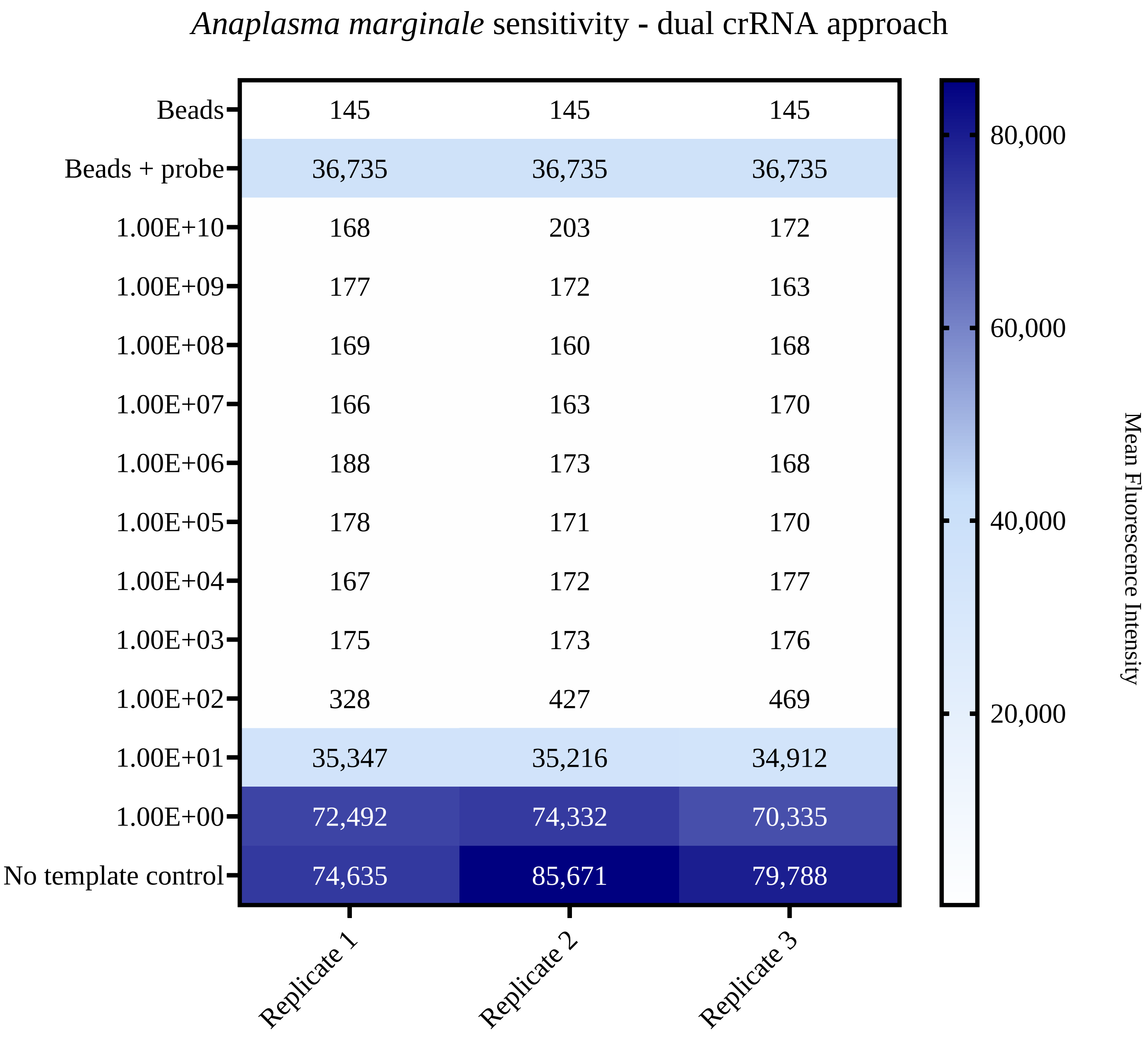

Sup. Fig. S4. Heat map representation of the mean fluorescence intensities for *Anaplasma marginale* sensitivity using a dual crRNA approach

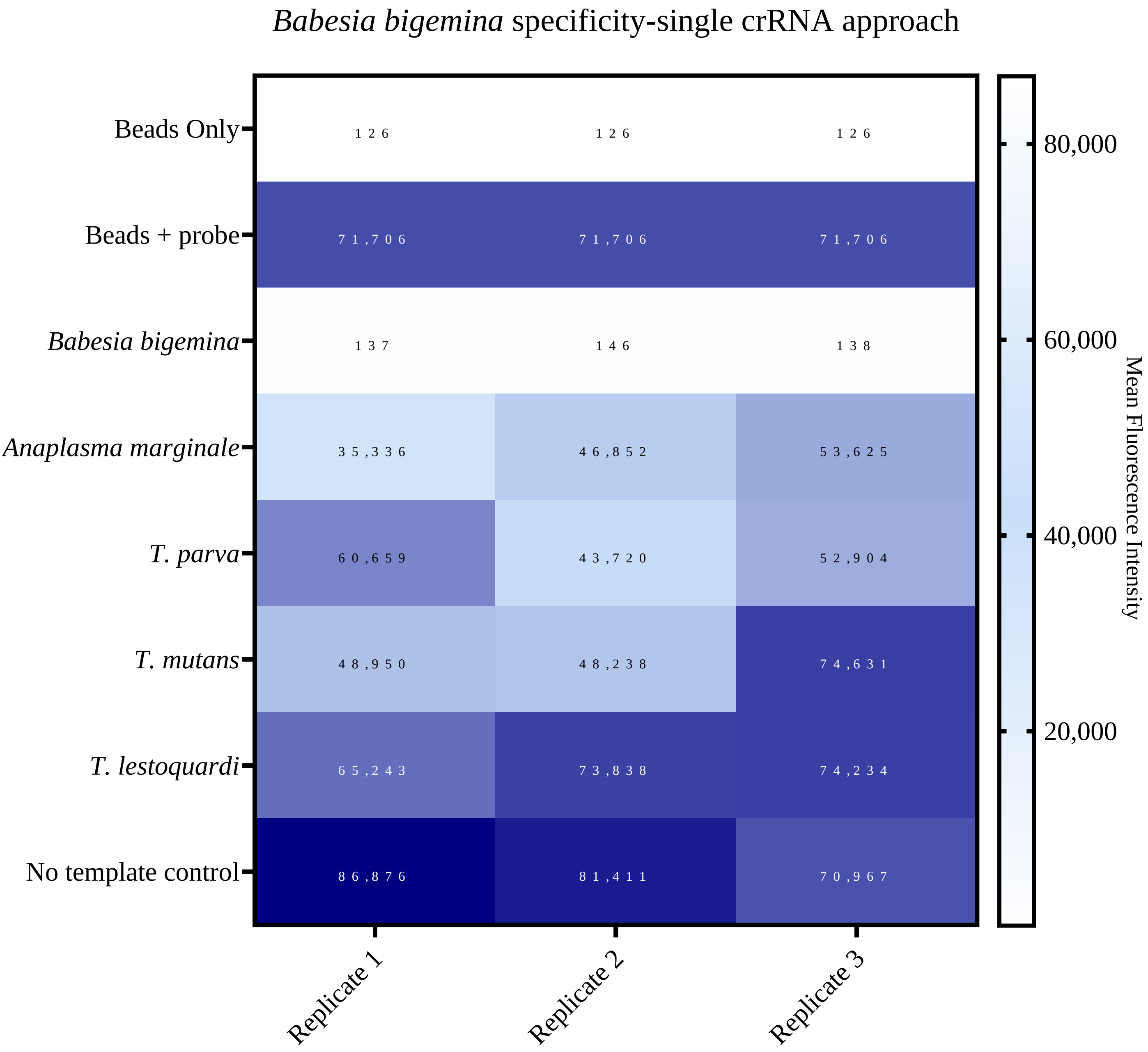

Sup. Fig. S5. Heat map representation of the mean fluorescence intensities for *Babesia bigemina* specificity using a single crRNA approach

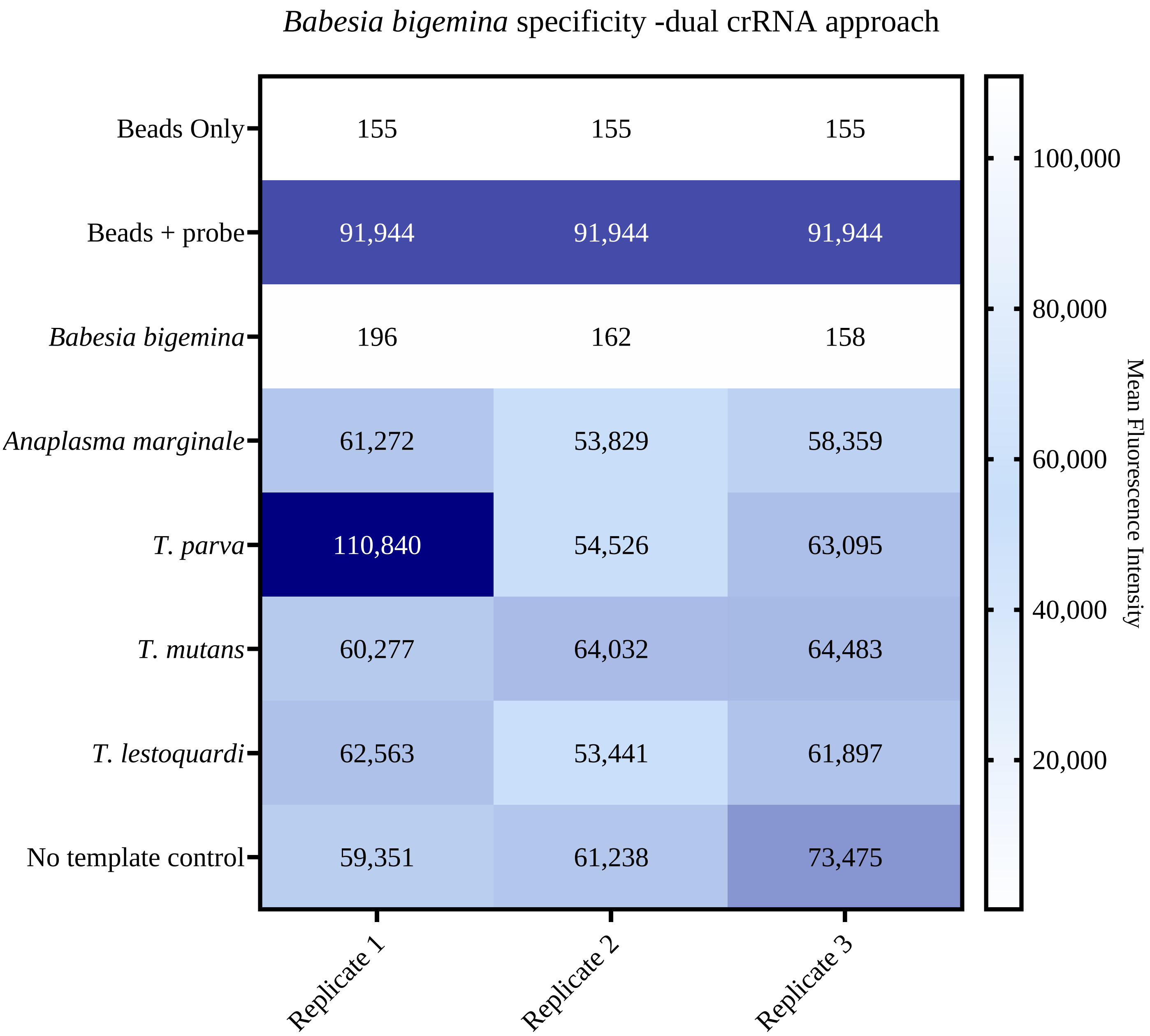

Sup. Fig. S6. Heat map representation of the mean fluorescence intensities for *Babesia bigemina* specificity using a dual crRNA approach

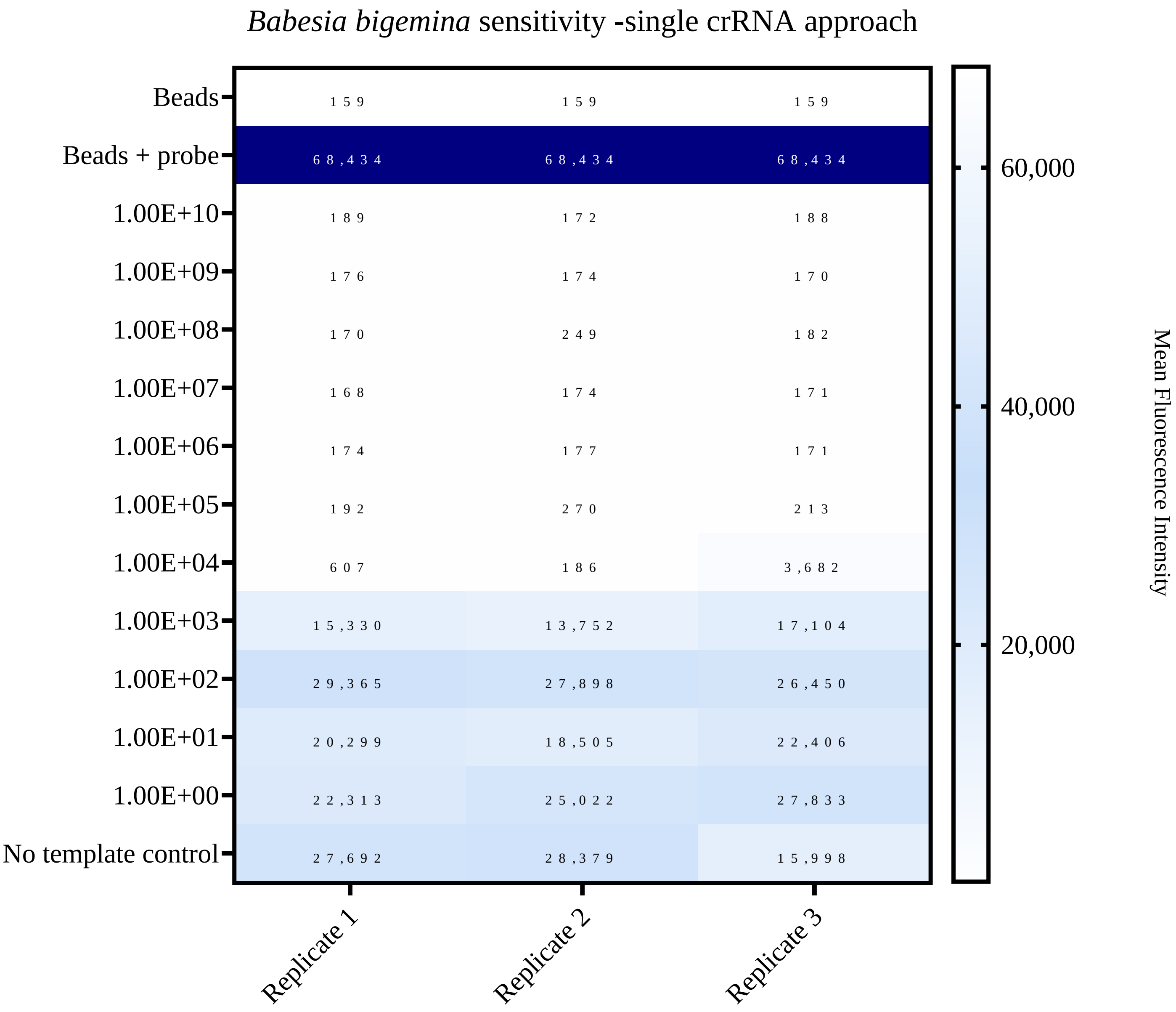

Sup. Fig. S7. Heat map representation of the mean fluorescence intensities for *Babesia bigemina* sensitivity using a single crRNA approach

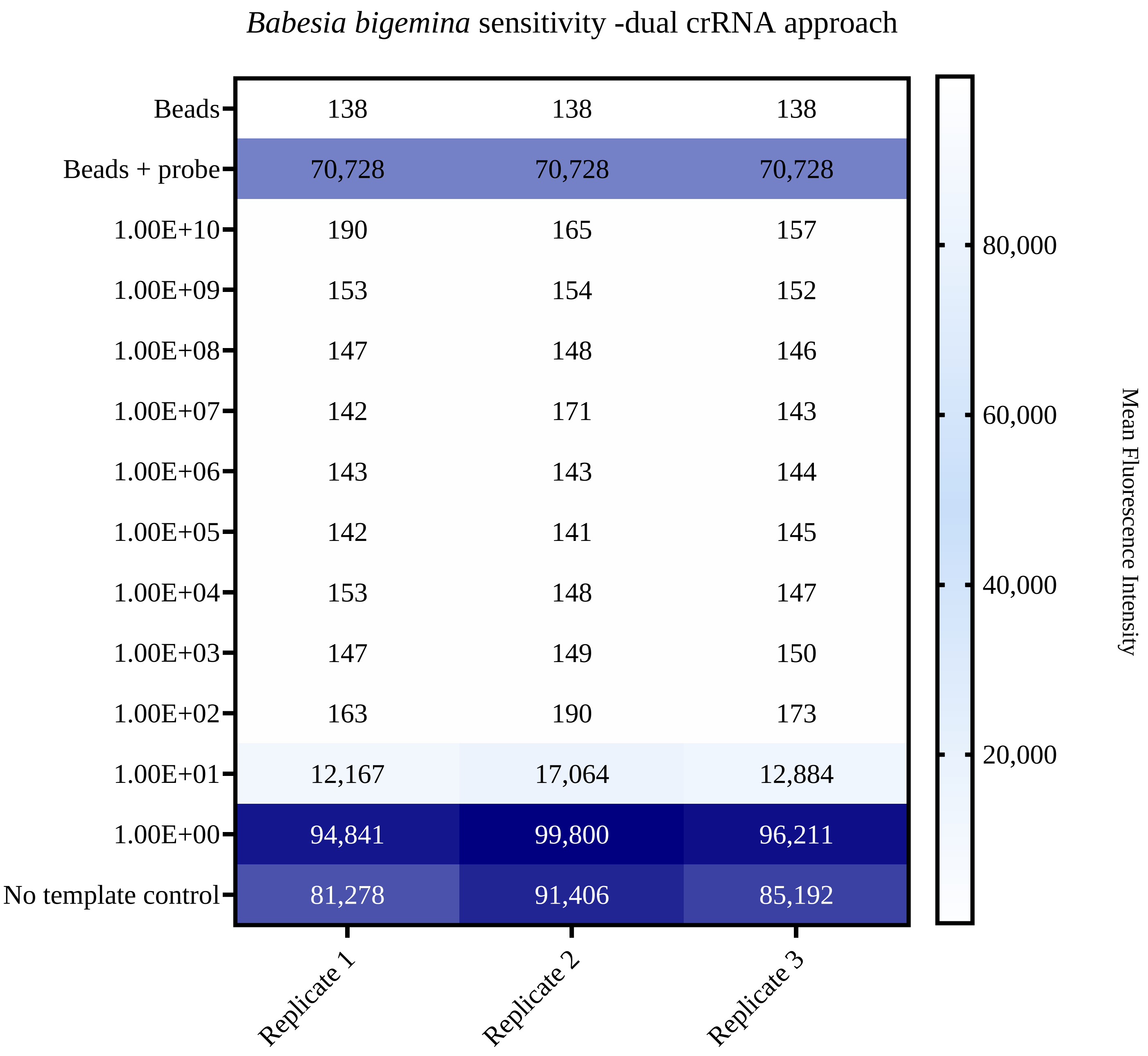

Sup. Fig. S8. Heat map representation of the mean fluorescence intensities for *Babesia bigemina* sensitivity using a dual crRNA approach
